# Enhanced Antidepressant-Like Effects of Electroacupuncture Combined with Citalopram in a Rat Model of Depression

**DOI:** 10.1155/2013/107380

**Published:** 2013-05-13

**Authors:** Jian Yang, Yu Pei, Yan-Li Pan, Jun Jia, Chen Shi, Yan Yu, Jia-Hui Deng, Bo Li, Xiao-Li Gong, Xuan Wang, Xiao-Min Wang, Xin Ma

**Affiliations:** ^1^Beijing Anding Hospital, Capital Medical University, 5 Ankang Alley, Beijing 100088, China; ^2^School of Arts and Law, Beijing University of Chemical Technology, Beijing 100029, China; ^3^Department of Physiology and Neurobiology, Capital Medical University, Key Laboratory for Neurodegenerative Disease of Education Ministry, 10 You An Men, Beijing 100069, China

## Abstract

Currently, antidepressants are the dominative treatment for depression, but they have limitations in efficacy and may even produce troublesome side effects. Electroacupuncture (EA) has been reported to have therapeutic benefits in the treatment of depressive disorders. The present study was conducted to determine whether EA could enhance the antidepressant efficacy of a low dose of citalopram (an SSRI antidepressant) in the chronic unpredictable stress-induced depression model rats. Here, we show that a combined treatment with 2 Hz EA and 5 mg/kg citalopram for three weeks induces a significant improvement in depressive-like symptoms as detected by sucrose preference test, open field test, and forced swimming test, whereas these effects were not observed with either of the treatments alone. Further investigations revealed that 2 Hz EA plus 5 mg/kg citalopram produced a remarkably increased expression of BDNF and its receptor TrkB in the hippocampus compared with those measured in the vehicle group. Our findings suggest that EA combined with a low dose of citalopram could produce greater therapeutic effects, thereby, predictive of a reduction in drug side effects.

## 1. Introduction

Depression is a common but serious mental disorder that affects more than 15% of the population during their lifetime [1]. Currently, antidepressants, especially selective serotonin reuptake inhibitors (SSRIs), are the mainstay in the treatment for depression. Unfortunately, many depressed patients do not respond well to presently available antidepressants and suffer from their severe side effects [2–5]. Therefore, it is necessary to seek complementary and alternative strategies with better efficacy of antidepressants and fewer side effects.

Acupuncture is a traditional complementary and alternative medicine approach that involves inserting fine needles into specific points to restore proper energy flow inside the body [6]. Electroacupuncture (EA) is a modification of acupuncture in which the needles inserted are attached with electrodes to deliver a pulsed electrical current. Numerous studies have demonstrated that acupuncture or EA treatment could alleviate depressive symptoms with very few side effects [7–10]. However, some researchers believe that there is a lack of sufficient evidence for supporting a beneficial effect from them [11, 12]. Although their utility in treating depression remains controversial, either of them may be considered as an adjunct to standard therapy [13, 14]. 

 Brain-derived neurotrophic factor (BDNF), a member of the neurotrophin family, plays critical roles in cell differentiation, neuronal survival, migration, and synaptic plasticity [15–17] and has been implicated in the pathophysiology of depression [18–21]. Postmortem studies have shown that hippocampal BDNF levels are decreased in depressed patients and increased in patients receiving antidepressant treatment [22–24]. Animal studies have demonstrated that various types of acute (e.g., immobilization and footshock) and chronic (e.g., chronic unpredictable stress (CUS) and chronic restrain) stress paradigms reduce BDNF expression in the hippocampus, and this reduction can be reversed by chronic antidepressant treatment [25–28]. Furthermore, several lines of evidence suggest that acupuncture or EA can upregulate hippocampal BDNF expression in normal and depression model rats [29–31].

Considering all of the aforementioned, the present study was designed to determine whether EA intervention combined with citalopram (a widely used SSRI), at a lower dose than what is required for monotherapy, could produce greater therapeutic effects in the CUS rats compared with EA or citalopram treatment alone. Changes in BDNF and its major receptor tropomyosin-related kinase receptor B (TrkB) were also evaluated.

## 2. Materials and Methods

### 2.1. Animals

Adult Sprague-Dawley (SD) rats (male, 200–220 g) were obtained from the laboratory animal center, Capital Medical University. Animals were maintained in a standard 12 h light/dark cycle in cages with ad libitum access to food and water and were allowed to acclimate to the environment for 7 days. All experimental procedures were approved by the Ethics Committee on Animal Care and Usage of Capital Medical University. Every effort was made to minimize animal suffering.

### 2.2. Experimental Design and Animal Groups

**Figure 1:**
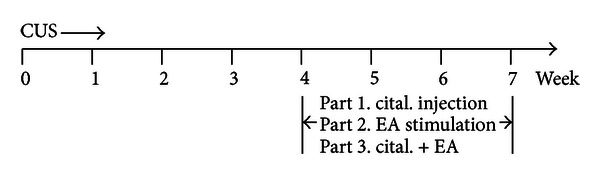
Schematic representation of the experimental procedure.

Rats in the control group received no CUS during the whole experiment. In the model group, rats were exposed to CUS for 7 weeks. From the beginning of the 5th week, model rats were randomly grouped and subjected to different experiments. In the first experiment, rats were divided into five groups and each group had 8-9 rats. Normal rats were intraperitoneally (i.p.) injected with saline, and model rats were, respectively, injected (i.p.) with saline and 5, 10, and 20 mg/kg citalopram once a day for 3 weeks. In the second experiment, there are four groups and each group had 8 rats. Normal rats received no treatment, while model rats were subjected to EA stimulation for 3 weeks and further divided into three groups: vehicle (received CUS only), 2 Hz EA, and 100 Hz EA. In the third experiment, model rats were administered with combined treatment with EA and citalopram for 3 weeks. In summary, there were five groups and each group had 8-9 rats: (1) vehicle (received CUS only); (2) 2 Hz EA group; (3) Cital 5 group; (4) Cital 5 plus 2 Hz EA group; (5) Cital 10 group. The experimental procedure is shown in Figure 1.

### 2.3. CUS Procedure

The CUS protocol was modified from the procedures described by Katz [32] and Willner et al. [33]. It consisted of a variety of sequential stressors applied randomly every day for 7 weeks (Table 1). Rats in the nonstress group were housed in groups of three, unless when they were subjected to sucrose preference test. Those exposed to CUS procedure were housed alone, unless when they were subjected to high-density housing. 

### 2.4. Sucrose Preference Test

The sucrose preference test was performed as previously described with minor modification [34, 35]. This test was carried out before CUS and at the end of each week. Rats were kept individually in separate cages and habituated to two needleless syringes (resp., filled with plain water and 1% sucrose solution) 8 h per day for 2 days. Followed by 12 h period of food and water deprivation, the rats were exposed to the two syringes for 30 min. After an interval of 1 h, the positions of the syringes were exchanged, and the rats were tested for 30 min again. The volume of sucrose solution and water consumption during the total 1 h test was recorded. Sucrose preference was expressed as a ratio of the volume of sucrose solution consumption to the volume of total fluid intake.

### 2.5. Open Field Test (OFT)

Locomotor activity was monitored automatically with infrared beams (each beam space 2.5 cm) in a black chamber before CUS and at the end of 4th and 7th weeks during the CUS procedure (TruScan 2.0 Instruments, Columbus, OH.). Tests were conducted between 9 a.m. and 11 a.m.. Each rat was placed in a corner of the chamber and was allowed to explore freely for 5 min. At the end of each test, the chamber was cleaned with 70% ethanol solution to remove any olfactory cues. Locomotor activity was defined as horizontal (floor plane, FP) and vertical (vertical plane, VP) movement distances and the number of entries into the arena-center (defined as more than 2.5-beam spaces away from the arena walls). Movement distances provide information on general activity. Number of center entries probably reflects anxiety-like behavior, with more “anxious” rats entering fewer times into the arena-center. The data were recorded and analyzed using a DigiScan analyzer and software (TruScan 2.0, Columbus). 

### 2.6. Forced Swimming Test (FST)

FST was performed at the end of 4th and 7th weeks during the CUS procedure. This test consists of a 15 min pretest swim and a 5 min test swim on the following day [36]. Rats were forced to swim in a glass cylinder (diameter 26 cm and height 60 cm) containing 35 cm depth of water at a temperature of 25°C. Water was changed after each test. Behavior was video-recorded using SMART video-tracking system (Panlab, Spain). Three types of behavior were analyzed: immobility, swimming, and climbing [37]. Immobility was defined as floating with no active activity other than those necessary to keep head above the water. Swimming was defined as active movements throughout the cylinder, including crossing into another quadrant. Climbing was defined as upward-directed movements of the forepaws against the cylinder walls.

### 2.7. Citalopram Treatment

Citalopram hydrobromide (Sigma-Aldrich, MO, USA) was dissolved in 0.9% physiological saline immediately before use and administered intraperitoneally daily from the 5th week to the 7th week during the CUS procedure. Control rats received saline as a vehicle injection.

### 2.8. EA Treatment

EA stimulation was administered from the 5th week following the CUS procedure. Two stainless steel needles of 0.25 mm in diameter were inserted at a depth of 5 mm into the acupoints of BAIHUI (GV 20, at the midpoint between the auricular apices) and Yintang (EX-HN 3, at the midpoint between the eyebrows). The bidirectional square-wave (0.2 ms) electrical pulse from a medical EA apparatus (HANS LY-257, Beijing) was administered with frequency 2 or 100 Hz for a total of 30 min each day, 6 days per week. The intensity of the stimulation was increased stepwise from 1 to 3 mA. During EA stimulation, the rats were kept under awake and unrestrained conditions in individual cages.

### 2.9. BDNF Protein Detection

Four to five rats in each group were killed by decapitation one day after behavioral measurements. Hippocampal tissue was dissected for protein assays and trunk blood was collected for the determination of serum BDNF levels. Samples were stored at −80°C until assay. Each frozen hippocampus was homogenized and lysed with RIPA buffer containing protease inhibitor cocktail (Sigma-Aldrich). The total protein concentration was determined using BCA protein assay kit (Pierce, Rockford, IL). For Western blot analysis, equal amounts of proteins were separated by 12% SDS-PAGE and transferred to PVDF (polyvinyldifluoride) membranes (Millipore). After being blocked with 5% nonfat-dried milk for 1 h, the membranes were incubated overnight at 4°C with rabbit polyclonal antibody to BDNF (1 : 300, Santa Cruz), rabbit polyclonal antibody to TrkB (1 : 1000, Millipore), and mouse monoclonal antibody to *β*-actin (1 : 5000, Sigma-Aldrich). Then the membranes were incubated with IRDye 800 conjugated secondary antibodies (Rockland Immunochemicals). Signals were visualized by the Odyssey infrared double-fluorescence imaging system (American Company LI-COR Biosciences, Lincoln, NE, USA). For enzyme-linked immunoassay (ELISA), hippocampal and serum BDNF levels were analyzed using a Chemokine BDNF ELISA kit according to the manufacturer's protocol (Millipore, Billerica, MA). The optical density was measured at 450 nm using an ELISA reader (Bio-Rad Laboratories Ltd, CA). ELISA results were expressed as ng per mL serum and pg per mg protein. All samples were assayed in duplicate. 

### 2.10. Statistical Analysis

Data were presented as means ± SEM. Statistical significance was assessed with the Student's *t*-test or one-way analysis of variance (ANOVA) followed by Newman-Keuls as post hoc multiple comparisons test using Prism 5.0 software (GraphPad Software). *P* value less than 0.05 was considered to be statistically significant.

## 3. Results

### 3.1. Chronic Unpredictable Stress Model

**Figure 2:**
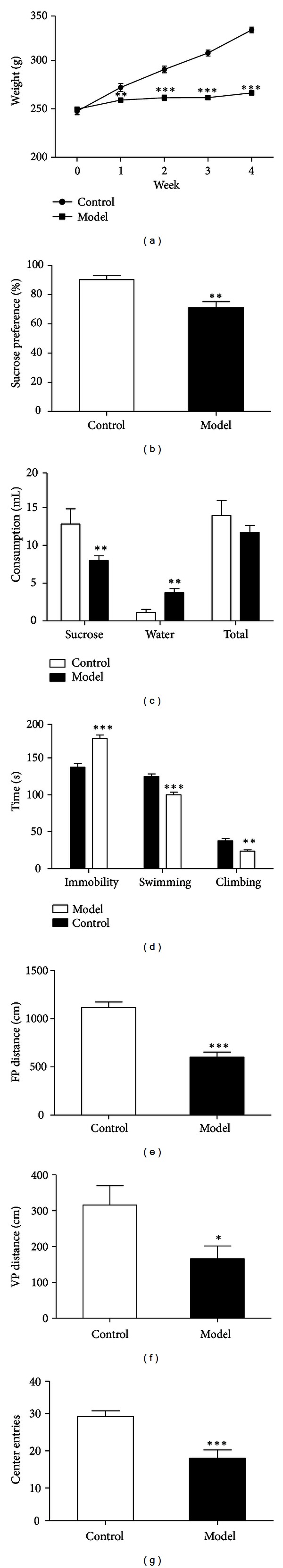
Rats show depressive-like symptoms after 4 weeks of CUS procedure. (a) Body weight. (b) Sucrose preference expressed as a ratio of the volume of sucrose solution consumption to the volume of total fluid intake. (c) The volume of consumption of sucrose solution, water, and total fluid. (d) FST. Data (expressed in seconds) are presented as time spent in immobility, climbing, and swimming. (e)–(g) OFT. Data (expressed in centimeter) are presented as movement distances in the floor plane (FP) (e) and vertical plane (VP) (f) and the number of entries into the arena-center (g). *n* = 11–15 per group. Data represent mean ± SEM, *P < 0.05, **P < 0.01, and ***P < 0.001  *versus* the control group.

Rats subjected to CUS exhibited significantly lower body weight compared with nonstressed controls from the first week after the commencement of CUS procedure (Week 1: control: 272.0 ± 4.028 g, CUS: 259.0 ± 2.152 g; Week 2: control: 290.6 ± 3.461 g, CUS: 261.4 ± 2.824 g; Week 3: control: 307.5 ± 3.090 g, CUS: 261.6 ± 1.946 g; Week 4: control: 331.3 ± 2.916 g, CUS: 266.4 ± 2.386 g.) (Figure 2(a)). Decreased sucrose preference is considered as a symptom resembling anhedonia, which is a core clinical feature of depression in humans [38, 39]. After 4 weeks of CUS, there was a significant decrease in sucrose preference in the model rats as compared with the controls, while the total fluid intake was not affected (Figures 2(b) and 2(c)). In the FST, the model rats demonstrated a significant increase in immobility time, accompanied by remarkably decreased swimming and climbing time (Figure 2(d)). Moreover, when exposed to the OFT, those rats showed significant decreases in the horizontal and vertical movement distances and fewer times into the arena-center (Figures 2(e)–2(g)). Taken together, these results indicate that the 4 weeks of CUS procedure is able to induce depressive-like symptoms (i.e., anhedonia, behavioral despair, and reduced locomotor activity) in the model rats.

### 3.2. Chronic Administration of Citalopram Ameliorates Depressive-Like Behavior

**Figure 3:**
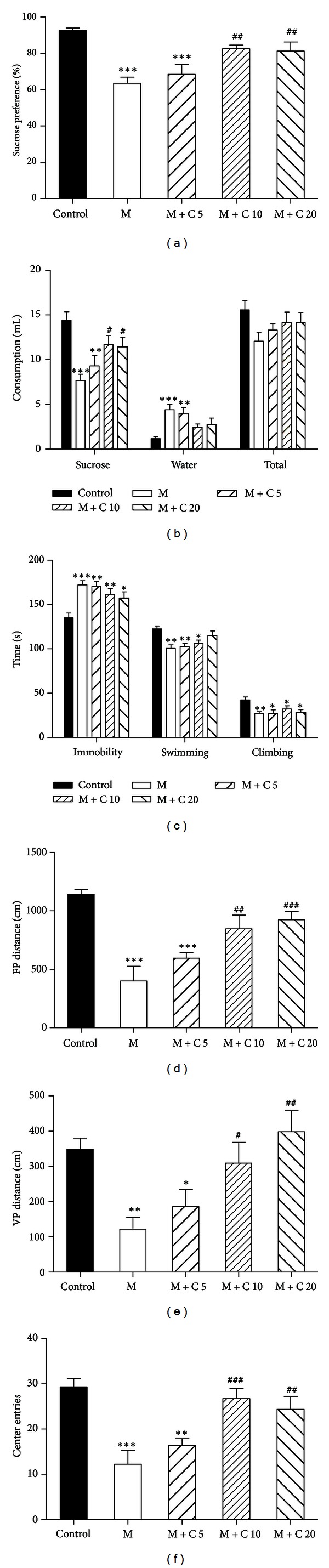
Effects of 3 weeks of treatment with citalopram on CUS-induced depressive-like behavior. (a) Citalopram significantly increased sucrose preference at 10 and 20 mg/kg but not 5 mg/kg. (b) Citalopram remarkably increased sucrose intake without affecting the total fluid intake. (c) Citalopram at different doses (5, 10, and 20 mg/kg) had no effect on immobility, climbing, and swimming in the FST. (d)–(f) In the OFT, citalopram significantly increased horizontal (d) and vertical (e) movement distances and center entries (f) at 10 and 20 mg/kg but not 5 mg/kg. *n* = 8-9 per group. Data represent mean ± SEM, *P < 0.05, and **P < 0.01  *versus* the vehicle-treated group.

Model rats were intraperitoneally injected with different doses of citalopram from the 5th week of CUS procedure. After 3 weeks of administration, both 10 and 20 mg/kg citalopram produced significantly increased sucrose preference, without affecting the total fluid intake (Figures 3(a) and 3(b)). In addition, they induced dramatic increases in horizontal and vertical movement distances (Figures 3(d) and 3(e)) and center entries (Figure 3(f)) in the OFT. However, these effects were not detected by treatment with 5 mg/kg citalopram. Moreover, none of these three doses of citalopram exerted any notable effect in the FST (Figure 3(c)). Thus, 5 mg/kg citalopram was considered ineffective and would be chosen to be combined with EA for further investigation.

### 3.3. EA Treatment Alone Only Improves Locomotor Activity of CUS Rats

**Figure 4:**
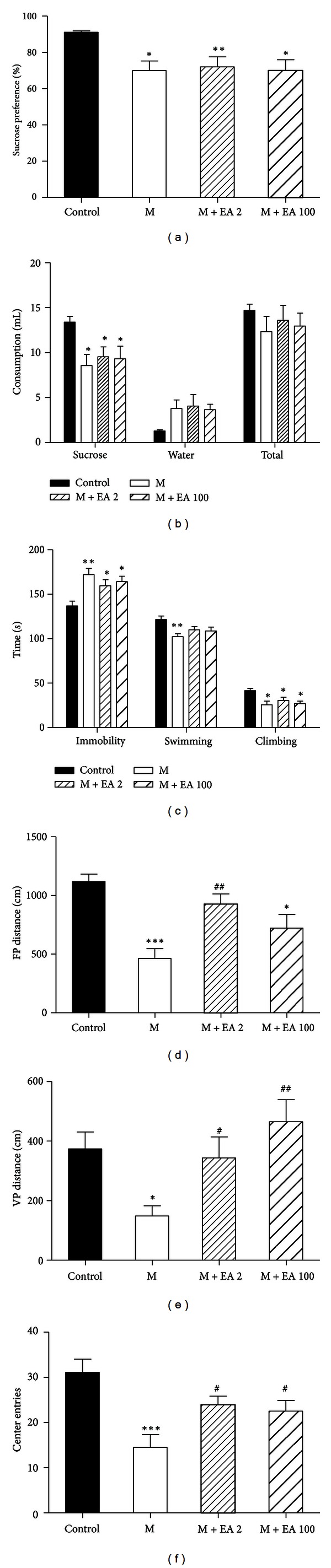
Effects of 3 weeks of EA stimulation on CUS-induced depressive-like behavior. (a)-(b) 2 Hz or 100 Hz EA stimulation had no effect on sucrose preference and sucrose intake. (c) Neither 2 Hz nor 100 Hz EA had any effect on immobility, climbing, and swimming in the FST. (d)–(f) In the OFT, 2 Hz EA induced significantly increased horizontal (d) and vertical (e) movement distances and center entries (f), while 100 Hz EA demonstrated no effect on the horizontal movement distance. *n* = 8 per group. Data represent mean ± SEM, *P < 0.05, and **P < 0.01  *versus* the vehicle-treated group.

 To assess whether EA could improve depressive-like behavior, model rats were administered with frequency 2 or 100 Hz EA stimulation from the 5th week of CUS procedure. After 3 weeks of treatment, neither 2 nor 100 Hz EA stimulation was able to improve anhedonia and behavioral despair as detected by sucrose preference test and FST (Figures 4(a)–4(c)). By contrast, both 2 and 100 Hz EA stimulation significantly increased vertical movement distance and center entries in the OFT, but only 2 Hz EA stimulation demonstrated a significant effect on the horizontal movement distance (Figures 4(d)–4(f)). Thus, we next explored the joint effects of 2 Hz EA and 5 mg/kg citalopram in the subsequent experiment.

### 3.4. Enhanced Antidepressant Effects of Combined Treatment with EA and Citalopram

**Figure 5:**
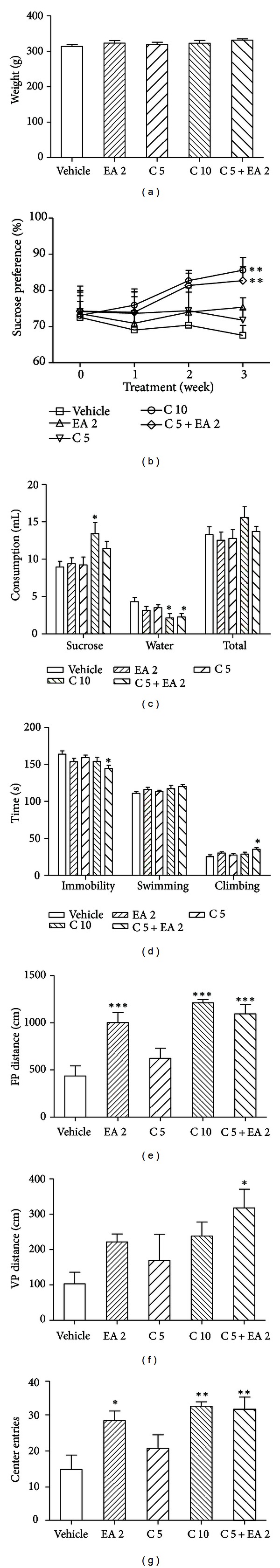
Effects of 3 weeks of combined treatment with EA and citalopram on CUS-induced depressive-like behavior. (a) Body weight. (b)-(c) 2 Hz EA plus 5 mg/kg citalopram as well as 10 mg/kg citalopram produced a substantial increase in sucrose preference without affecting the total fluid intake. (d) 2 Hz EA plus 5 mg/kg citalopram induced significantly decreased immobility time and increased climbing time in the FST. (e)–(g) 2 Hz EA plus 5 mg/kg citalopram led to significantly increased horizontal (e) and vertical (f) movement distances and center entries (g). *n* = 8-9 per group. Data represent mean ± SEM, *P < 0.05, **P < 0.01, and ***P < 0.001  *versus* the vehicle-treated group.

To evaluate whether EA combined with citalopram has an additive or synergistic antidepressant effect, combined treatment with 2 Hz EA and a low dose of citalopram was administered from the 5th week of CUS procedure. After 3 weeks, 2 Hz EA plus 5 mg/kg citalopram led to substantial increases in sucrose preference (Figures 5(b) and 5(c)), horizontal/vertical movement distances (Figures 5(e) and 5(f)), and center entries (Figure 5(g)), demonstrating similar effects to 10 mg/kg citalopram. Furthermore, 2 Hz EA plus 5 mg/kg citalopram induced a significant reduction in immobility time, accompanied by an increased climbing time in the FST, whereas these effects were not detected by treatment with 10 mg/kg citalopram (Figure 5(d)). However, there was no remarkable difference in body weight among all the groups (Figure 5(a)). These findings suggest that 2 Hz EA plus 5 mg/kg citalopram could exert better antidepressant effects in comparison with either treatment alone.

### 3.5. Combined Treatment with EA and Citalopram Induces a Higher Expression of BDNF in the Hippocampus

**Figure 6:**
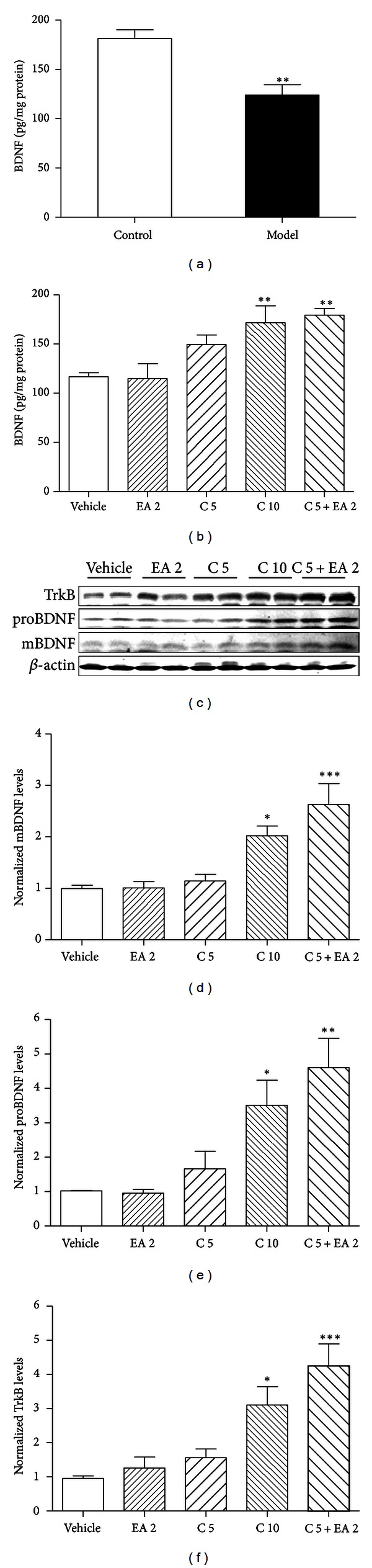
CUS-induced downregulation of BDNF expression was reversed by 3 weeks of combined treatment with EA and citalopram. (a) Hippocampal BDNF protein levels were significantly decreased after 4 weeks of CUS procedure. (b) ELISA results showed that 2 Hz EA plus 5 mg/kg citalopram as well as 10 mg/kg citalopram induced remarkably increased BDNF protein levels in the hippocampus. (c) Western blot analysis of hippocampal mBDNF, proBDNF, and TrkB protein levels. *β*-actin was used as an internal control. (d)–(f) Quantitative analysis of mBDNF, proBDNF, and TrkB protein levels in (b). *n* = 4-5 per group. Data represent mean ± SEM, *P < 0.05, **P < 0.01, and ***P < 0.001  *versus* the vehicle-treated group.

To explore the possible mechanisms involved in the joint effects of EA and citalopram, the rats were sacrificed one day after behavioral tests. Results from ELISA demonstrated that hippocampal BDNF protein levels were significantly decreased after 4 weeks of CUS procedure (Figure 6(a)). However, 3 weeks of treatment with 2 Hz EA plus 5 mg/kg citalopram, as well as 10 mg/kg citalopram, led to a remarkable increase in BDNF expression in the hippocampus (Figure 6(b)), but not in the prefrontal cortex or serum (data not shown). Western blot analysis also showed that protein levels of both mature BDNF (mBDNF) and BDNF precursor (proBDNF) significantly increased in 2 Hz EA plus 5 mg/kg citalopram group, similar to the observations in 10 mg/kg citalopram group. Moreover, both of these groups revealed a dramatic increase in the expression of TrkB compared with that measured in the vehicle group (Figure 6(c)). These data indicate that EA combined with citalopram could prevent CUS-induced decrease in BDNF signaling in the hippocampus.

## 4. Discussion

The present study aimed at exploring the therapeutic potentiality of coadministration of EA and an antidepressant by biochemical and behavioral approaches using an animal model predictive of antidepressant-like activity. Here, we demonstrated that the combined treatment with 2 Hz EA and a low dose of citalopram could prevent CUS-induced decrease in hippocampal BDNF signaling and exert better antidepressant effects in the CUS model rats than either treatment alone. 

CUS model, which mimics socioenvironmental stressors in everyday life, is one of the most extensively used animal models of depression [40]. Rats subjected to the CUS paradigm for several weeks can exhibit almost all demonstrable depressive symptoms. However, this model is difficult to replicate, because different CUS schedules, including types of stressors, animal strains, and nutritive status, can result in inconsistent findings [39, 41]. It has been reported that chronic restraint stress can induce significant downregulation of BDNF in the hippocampus [27, 42]. Accordingly, restraints were used frequently in our CUS paradigm. Consistent with previous studies, we have demonstrated that rats subjected to CUS for 4 weeks exhibit a significantly decreased sucrose preference, accompanied by other behavioral changes such as increased immobility time and decreased locomotor activity. 

FST is widely used as a screening procedure for antidepressants [37, 43]. In this test, animals display “despair” behavior (immobility) and active behaviors (swimming and climbing) (14). It has been demonstrated that different SSRIs may exert different effects on immobility time [44, 45]. In our experiment, citalopram was demonstrated inactive after chronic administration in the FST, consistent with several previous researches [46, 47]. On the contrary, many other studies have shown positive effects of acute administration of citalopram on immobility and swimming time in acute stress models [48–50]. The missing observation of significant changes in our experiment might be attributed to two main causes. One is the rat strain. It has been shown that SD rats respond to various antidepressants not so well as Wistar-Kyoto rats in the FST [44]. The other is the type of stressors. Repeated swimming stress may depress the sensitivity of model rats to FST. Also, it has been reported that food restriction with modest weight reduction can attenuate the behavioral effects of SSRIs [51]. Even so, we demonstrated that high doses (10 and 20 mg/kg) of citalopram were able to result in significantly increased sucrose preference and improved locomotor activity in the model rats as previously reported [52, 53].

Acupuncture has been applied to treat depressive disorders with a long clinical history, but a recent Cochrane review identified that no consistent benefit was noted with any form of acupuncture (manual acupuncture, EA, or laser acupuncture) in depressed patients [6, 14]. In the present study, we found that sucrose preference and immobility time in the model rats were not significantly affected by EA treatment, even though there was a tendency of increase or reduction after 3 weeks of stimulation. These results are in agreement with several previous studies that were also performed on the CUS model rats [54–56]. Liu and his colleagues demonstrated that 3 weeks of EA stimulation produced a significant increase in the number of crossing in the OFT and a nonsignificant increase in the sucrose intake [55]. Another study reported by Yu et al. showed that sucrose preference and immobility time in the depressive rats were not significantly changed by 6 weeks of treatment with EA alone [56]. Because frequency is an important parameter involved in the efficiency of EA [57], we explored the effects of both high- (100 Hz) and low-frequency (2 Hz) EA in the present study. Although neither of them had a definite antidepressant-like effect, 2 Hz EA exerted a remarkable increase in the horizontal movement distance and a better tendency in sucrose preference and immobility time. Thus, we supposed that 2 Hz EA might be more suitable for combined treatment with a lower dose of citalopram.

As expected, we found that 2 Hz EA combined with a low dose of citalopram led to significant improvement in the sucrose preference test and FST, showing a greater effect on depressive-like behavior than either treatment alone. Similarly, several previous literatures have shown that EA augmented antidepressant-like effects of a tricyclic antidepressant, clomipramine or mianserin, in depression model rats or patients [54, 56, 58]. It has also been reported that application of acupuncture to low-dose fluoxetine-treated depressed patients is as effective as a recommended dose of fluoxetine treatment. However, the mechanisms underlying these combined effects remain unknown.

The delayed onset of SSRIs suggests that their therapeutic effects are mediated beyond a simple enhancement in serotonergic neurotransmission but may be more related to reorganization of neuronal networks [59]. Numerous studies have identified a key role of BDNF in the development and treatment of depression. BDNF needs binding to high-affinity protein kinase receptor family, TrkB, to exert its biological effects, such as neuronal survival, differentiation, neurotransmitter release, and synaptic plasticity. There is a clear evidence that BDNF-TrkB signaling mediates the actions of antidepressants [60]. For instance, chronic SSRI treatment could increase BDNF expression and TrkB receptor activation in rodent hippocampus [25, 61]. Consistent with these findings, the present study demonstrates that chronic administration of 10 mg/kg citalopram increased both BDNF and TrkB protein levels in rat hippocampus. Moreover, combined treatment with 2 Hz EA and 5 mg/kg citalopram, but not either treatment alone, produced similar effects, corresponding to improved depressive-like behavior. However, the relationship between the change of BDNF-TrkB signaling and the antidepressant-like effect of the combined therapy needs further investigation. 

In conclusion, our study points out that the combined treatment with EA and a low dose of citalopram could prevent CUS-induced decrease in hippocampal BDNF signaling and lead to greater antidepressant effects than either treatment alone. These findings could provide a new perspective on clinical therapy for depression.

## Figures and Tables

**Table 1:** Schedule of chronic mild stress procedures.

Day	Stressors	Time
1	10 h crowded cage (5-6 rats per cage)	a.m. to p.m.
2	15 min forced swim (22°C) 15 h wet bedding	p.m.
3	4 h restraint 24 h food deprivation	a.m.
4	5 min cold swim (4°C)	p.m.
5	4 h restraint 24 h water deprivation (including 6 h empty water bottle)	a.m.
6	2 h cold stress (15°C) 4 h restraint	a.m. p.m.
7	4 h restraint 2 min tail pinch	a.m. p.m.
